# Lichen Planus Pigmentosus and Vitiligo in a 61-Year-Old Filipino Man: Case Report

**DOI:** 10.2196/50401

**Published:** 2024-11-29

**Authors:** Maria Isabel Belizario, Julius Garcia Gatmaitan, Johannes Dayrit

**Affiliations:** 1 Belizario Dermatology Clinic, , Quezon City, Philippines; 2 Skines Aesthetic and Laser Center, Quezon City, Philippines; 3 Department of Dermatology, De La Salle Medical and Health Sciences Institute, Dasmariñas, Cavite, Philippines

**Keywords:** lichen planus pigmentosus, vitiligo, autoimmune, isotretinoin, tacrolimus, skin, melanin, hyperpigmentation, LPP

## Abstract

Pigmentary disorders have been implicated in causing psychosocial turmoil in patients as they can cause some degree of cosmetic disfigurement. Lichen planus pigmentosus (LPP) presents as ashy, dermatosis-like eruptions on sun-exposed areas, particularly on the head, neck, and earlobes. On the other hand, vitiligo is a chronic disorder that appears as depigmented patches on the skin. A 61-year-old man with Fitzpatrick skin phototype IV presented to us initially with LPP but eventually also developed vitiligo. The patient was treated with low-dose oral isotretinoin for LPP and topical tacrolimus 0.1% ointment for both LPP and vitiligo with a good clinical outcome. One case of segmental vitiligo and zosteriform LPP, affecting a 22-year-old Indian woman, has been previously reported in the English-language literature. An autoimmune etiology that causes melanocytorrhagy may be a plausible hypothesis for the coexistence of these 2 conditions.

## Introduction

### Overview

Lichen planus pigmentosus (LPP), first described by Bhutani et al in 1974, appears after the age of 30 and presents as ashy, dermatosis-like macules on sun-exposed areas that later merge to form hyperpigmented patches (either diffuse, reticulate, or perifollicular) [1,2]. Vitiligo is a rare, chronic disorder that appears in about 1% of the population, causing depigmentation of the skin without preceding inflammation [3]. Although clinically opposite in presentation, autoimmunity has been implicated in these 2 conditions [4].

Pigmentary disorders are a source of psychosocial turmoil and cosmetic disfigurement. The quality of life of patients with LPP and vitiligo was found to be more significantly affected than that of patients with melasma [5].

To the best of our knowledge, only 1 case of coexistent LPP and vitiligo has been reported in the English-language literature [6]. We present a case of a 61-year-old man who was diagnosed with LPP but later developed facial, nonsegmental vitiligo. The patient significantly improved following 4 months of low-dose oral isotretinoin for LPP and tacrolimus 0.1% ointment for both LPP and vitiligo.

### Ethical Considerations

This case report was obtained in private practice and is published for educational purposes rather than for research. Informed consent was obtained. Since there was no intervention given to the patient that deviated from standard practice, no institutional review board approval was required.

## Case Report

**Figure 1.**
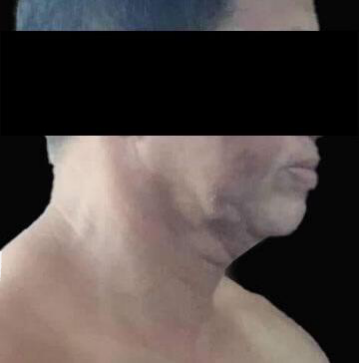
Clinical presentation of hyperpigmented patches: multiple, ill-defined, ashy gray macules and patches on the forehead, malar, nasal bridge, and perioral areas.

**Figure 2.**
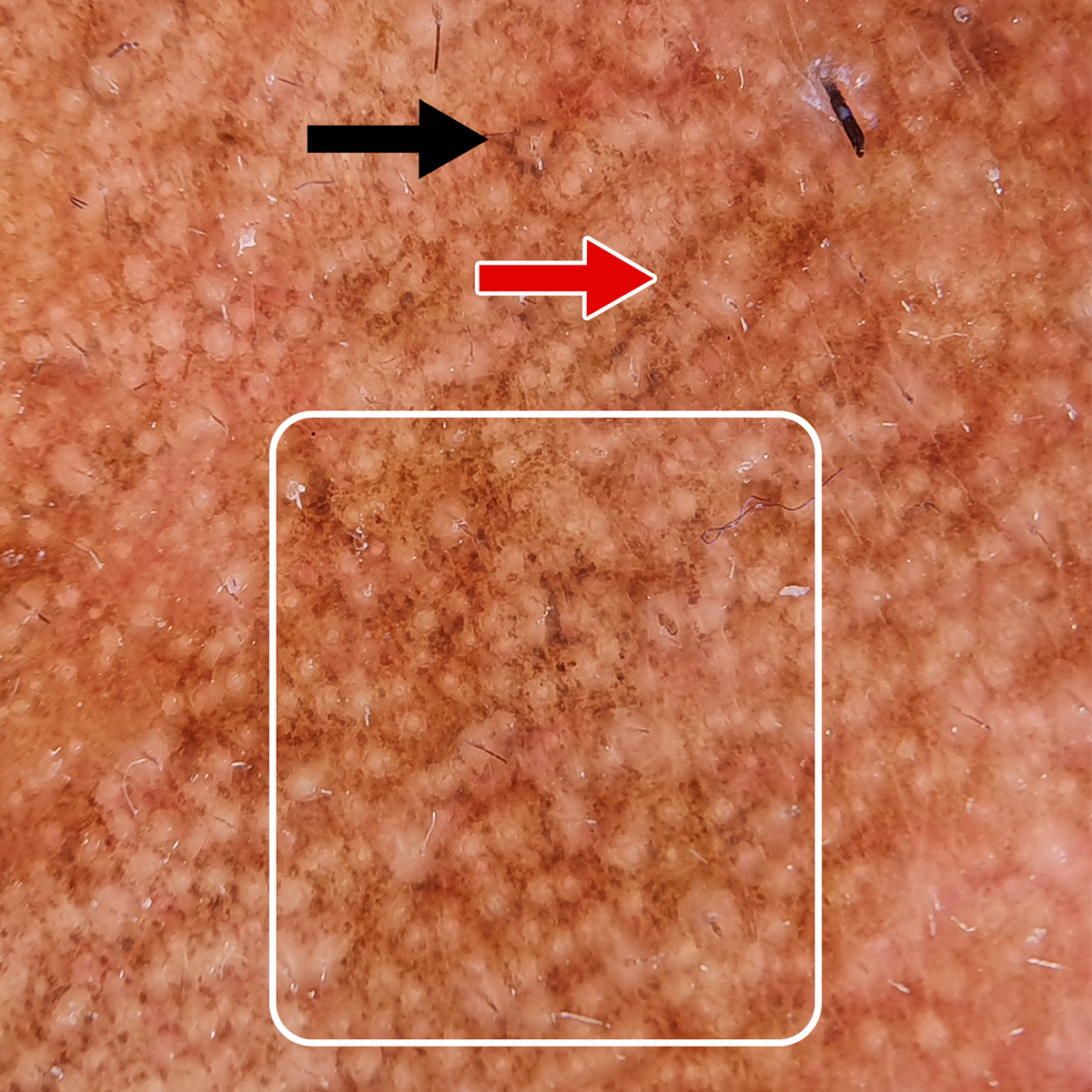
Dermoscopic findings (DermLite DL4 ×10) of hyperpigmented patches: dots (black arrow) in arcs, lines, and circles forming a hemlike pattern (red arrow), with larger areas reminiscent of marbled wagyu (white square).

**Figure 3.**
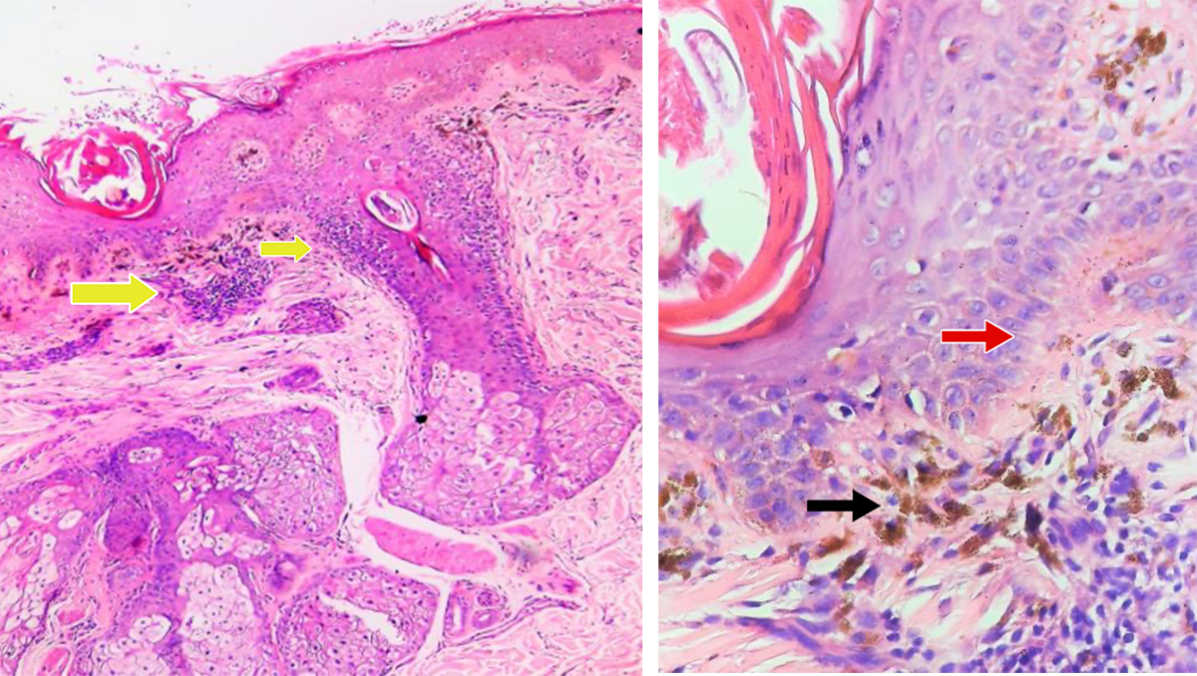
Histopathologic findings on a low power objective revealed a mild focally lichenoid and a superficial perivascular and periadnexal inflammatory infiltrate of lymphocytes (yellow arrows), while a high power objective (H&E ×1000) showed focal vacuolar alteration (red arrow) and large, pigment-laden macrophages (black arrow).

A 61-year-old man with Fitzpatrick skin phototype IV consulted us after developing hyperpigmented macules and patches on his malar area over 5 months. Lesions slowly increased in size and number, across the forehead, nasal bridge, and perioral areas. They were asymptomatic and aggravated by sun exposure. He had no history of drug intake or inflammatory dermatoses. Dermatological examination revealed multiple, ill-defined, ashy gray macules and patches on the face (Figure 1). A dermoscopy of the hyperpigmented areas revealed perifollicular dark-brown clods in arcs, lines, and circles. Larger areas formed a hemlike pattern and were reminiscent of marbled wagyu (Figure 2). A skin punch biopsy was obtained from the hyperpigmented area on the left cheek, after securing informed consent for the procedure and his case’s subsequent publication. Results showed a mild focally lichenoid and a superficial perivascular and periadnexal inflammatory infiltrate of lymphocytes. Further magnification revealed focal vacuolar alteration of the basal cell layer with numerous pigment-laden macrophages (Figure 3). The patient had negative antinuclear antibody test results and normal findings in the following parameters: complement component 3 (C3) blood test, chest x-ray, hepatitis B and C profile, 2D echocardiography, electrocardiogram, thyroid-stimulating hormone, free thyroxine, free triiodothyronine, and serum chemistry.

**Figure 4.**
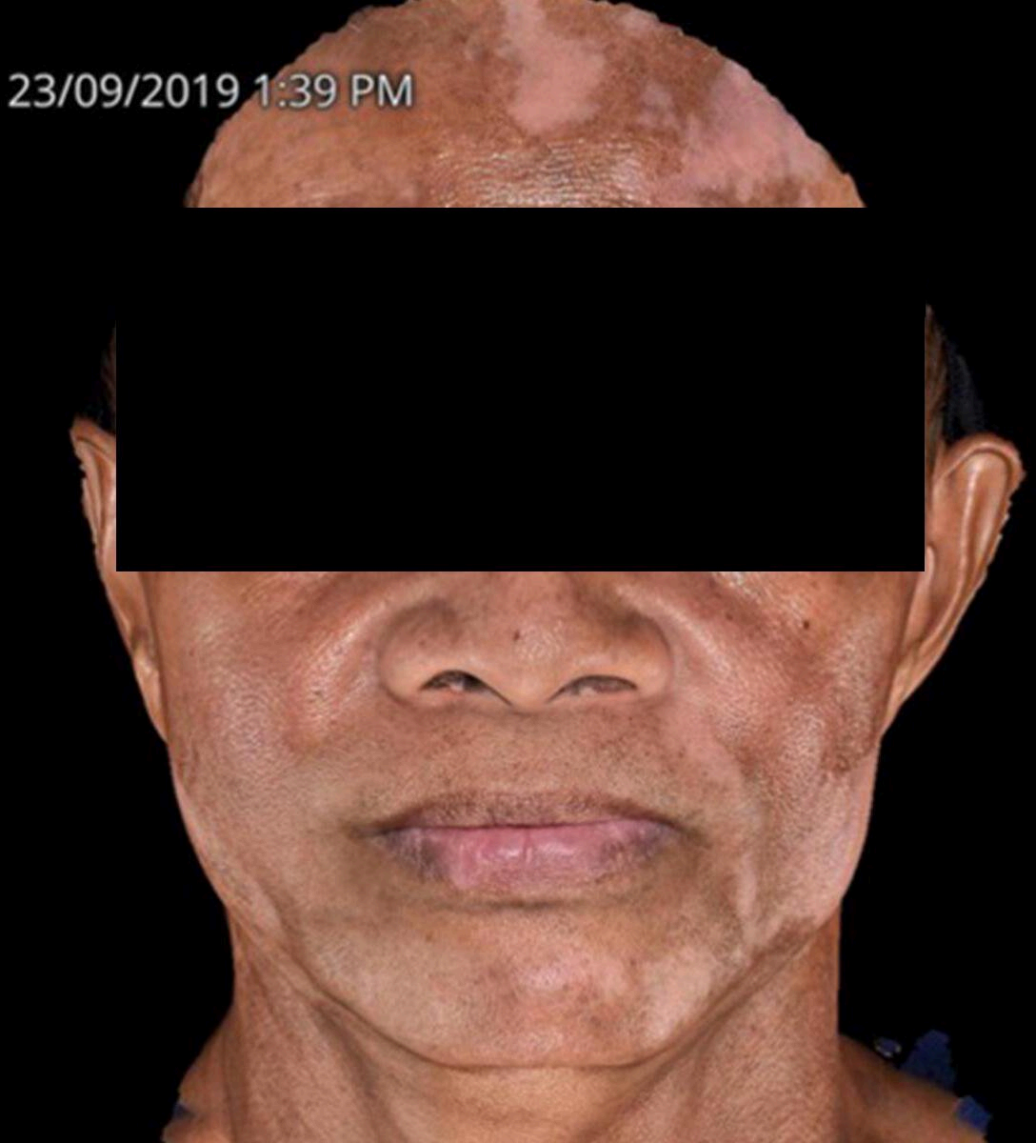
Patient diagnosed with lichen planus pigmentosus but now presenting with sharply demarcated, ill-defined depigmented (white) patches on both sides of the face.

**Figure 5.**
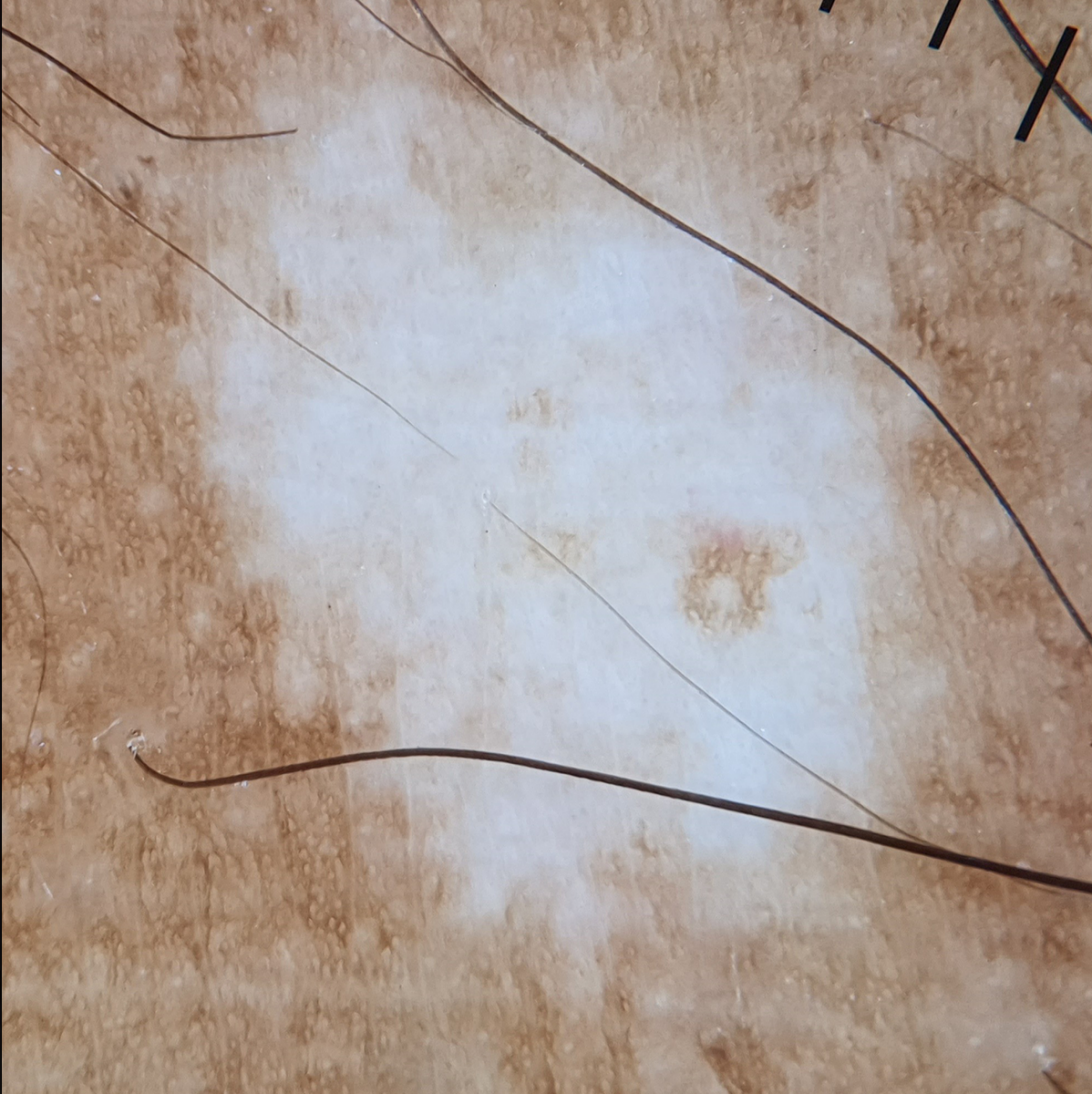
Dermoscopic findings (DermLite DL4 ×10) of depigmented patches: irregularly shaped white areas with a loss of skin markings and some with brown central areas (follicular repigmentation).

**Figure 6.**
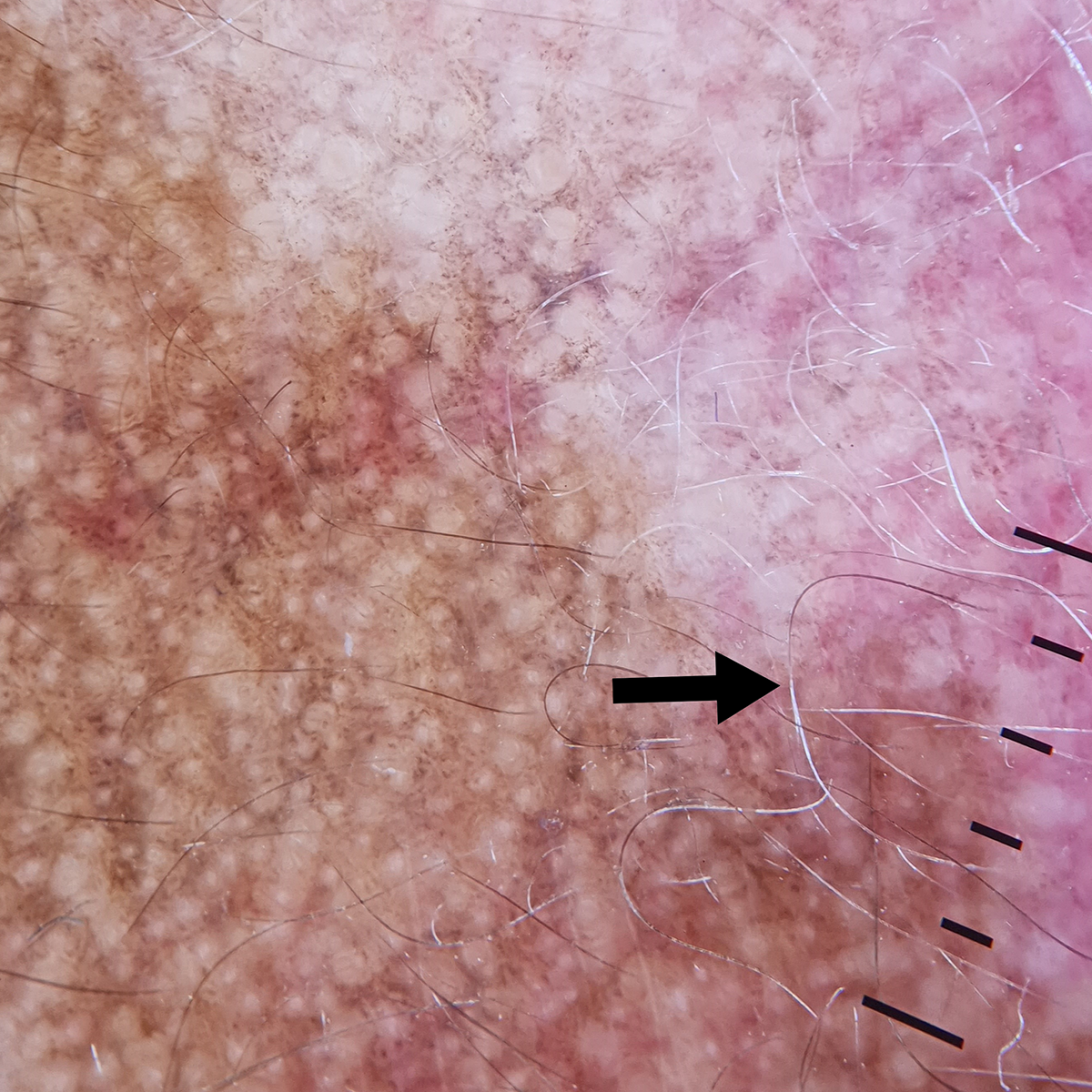
Dermoscopic findings (DermLite DL4 ×10) showed perifollicular black dots with black hairs and white areas with white hairs (black arrow). A transition zone was observed between the brown and white areas.

**Figure 7.**
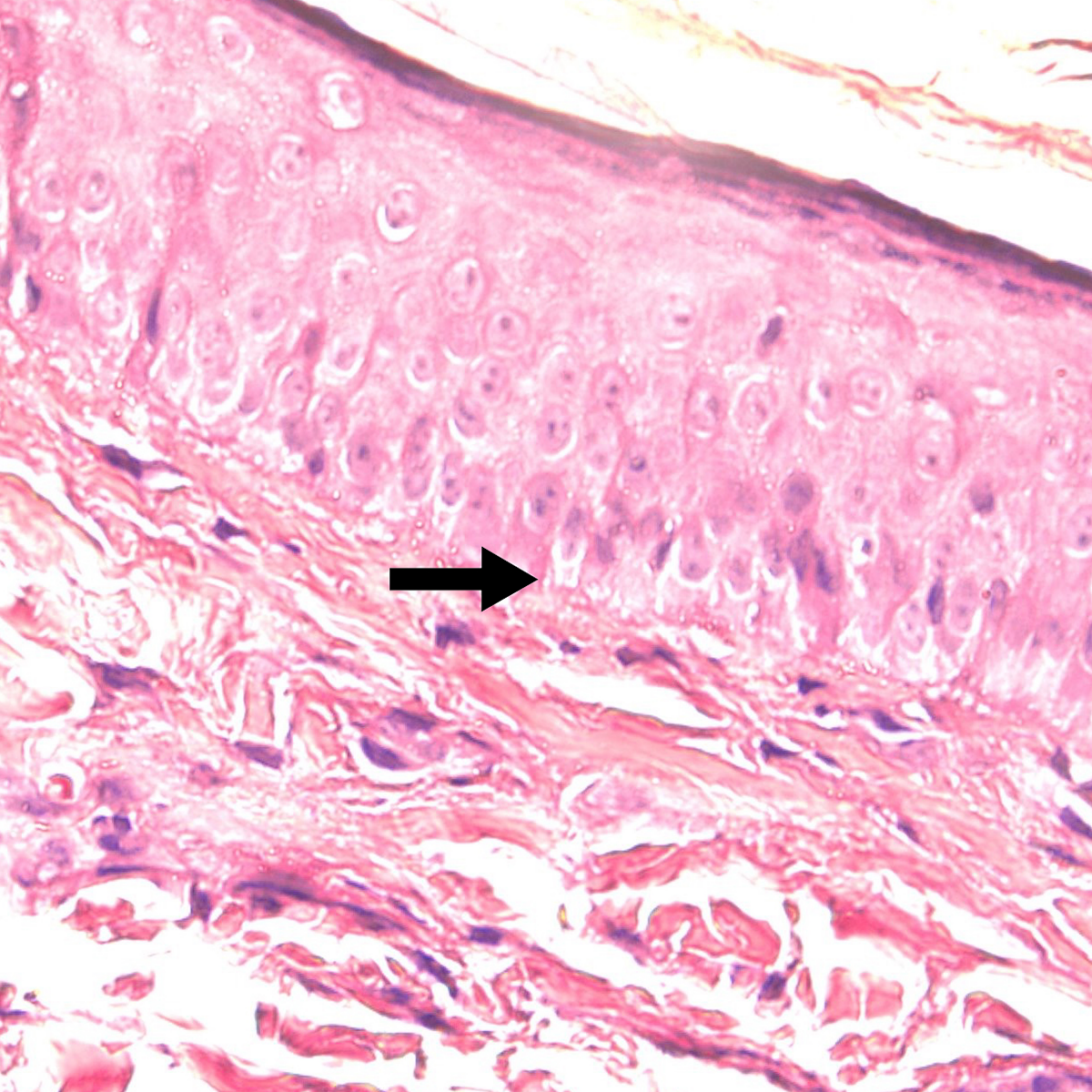
Histopathologic findings revealed the absence of melanocytes in the basal cell layer (black arrow) and a sparse, superficial perivascular inflammatory infiltrate of lymphocytes.

The patient was lost to follow-up before treatment was initiated. He returned after 1 year due to persisting hyperpigmentation and new, sharply demarcated, ill-defined depigmented patches on both sides of the face (Figure 4). Dermoscopies of the depigmented and hyperpigmented areas revealed irregularly shaped white areas with a loss of skin markings and follicular clods compatible with vitiligo (Figure 5) and perifollicular brown dots in arcs and circles (Figure 6). A transition zone was seen between the brown and white areas. A skin punch biopsy of the depigmented patch on the forehead showed the absence of melanocytes in the basal cell layer (Figure 7).

**Figure 8.**
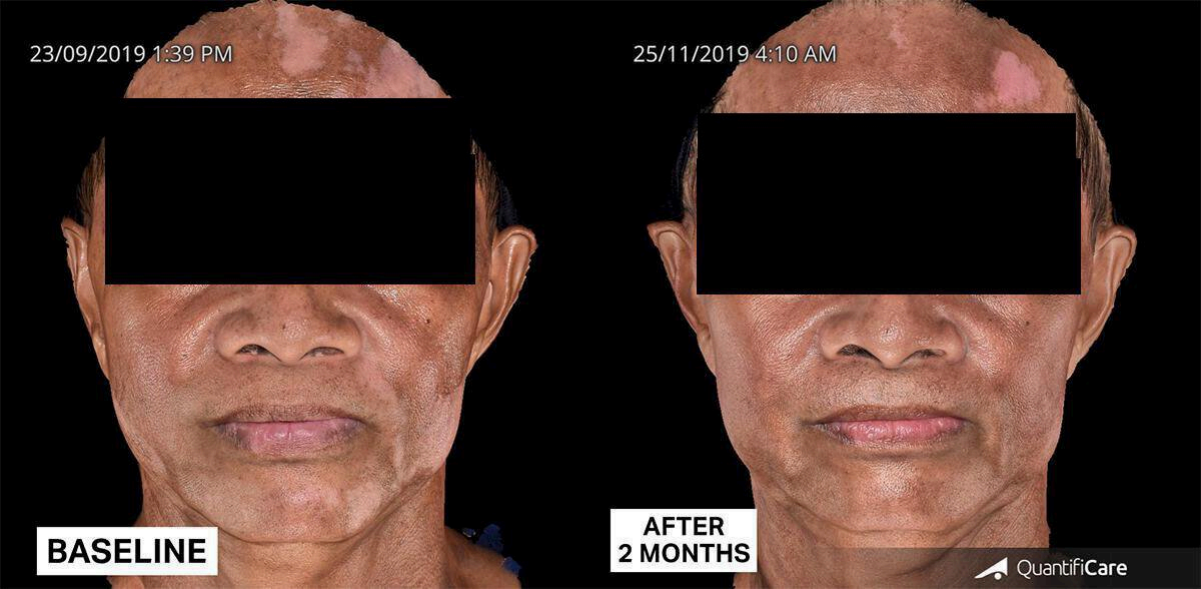
Patient after 2 months of continuous treatment with low-dose isotretinoin for lichen planus pigmentosus and tacrolimus 0.1% ointment for both lichen planus pigmentosus and vitiligo.

**Figure 9.**
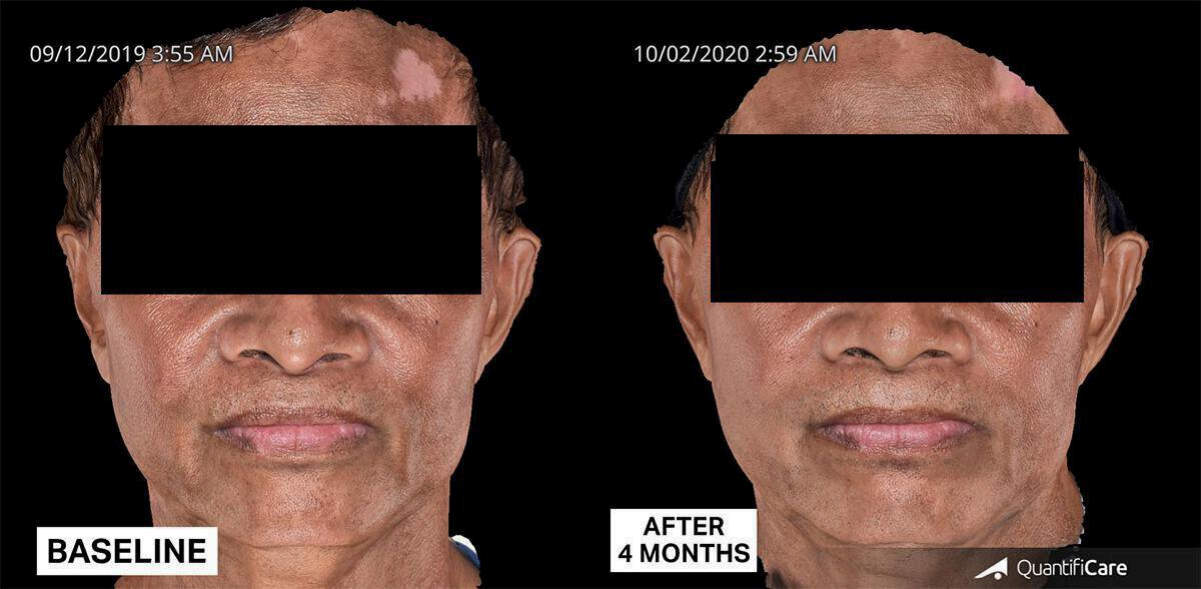
Patient at follow-up after 4 months of continuous treatment.

**Figure 10.**
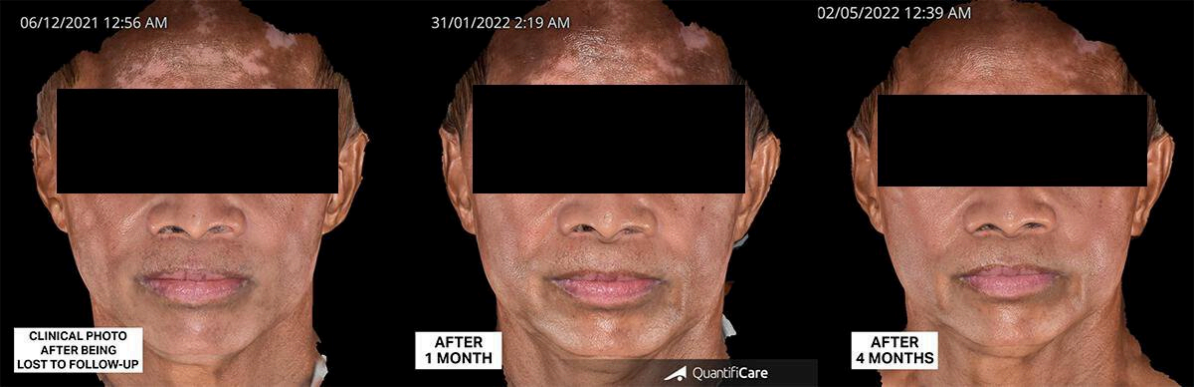
Clinical presentation post the COVID-19 pandemic, showing increased depigmented patches. Significant lightening of lichen planus pigmentosus and repigmentation of vitiligo were observed after 1 month and 4 months of continuous application of topical regimen with photoprotection.

The patient was finally diagnosed with LPP and facial, nonsegmental vitiligo, and started on low-dose isotretinoin at 10 mg once a day for 1 month, then 10 mg every other day for the second month. He was also prescribed topical tacrolimus 0.1% ointment twice a day and advised to use sunscreen daily. Follow-up showed lightening of the hyperpigmented areas by 60% and repigmentation of vitiliginous patches by 80% after 2 months of treatment (Figure 8), and lightening by 80% and repigmentation by 90% after 4 months (Figure 9). Isotretinoin was discontinued. However, due to the COVID-19 pandemic, the patient was again lost to follow-up for 1 year and became noncompliant with the given treatments. Upon return to the clinic, more vitiliginous lesions were observed, but facial hyperpigmentation had significantly improved. At this time, the patient was advised to resume daily use of topical tacrolimus 0.1% ointment and broad-spectrum sunscreen. Progressive improvement was seen after continuous application of a prescribed topical regimen after 1 month (80%) and 4 months (90%) (Figure 10).

## Discussion

The exact pathogenesis of LPP remains unknown. However, UV exposure and certain viral infections and cosmetics have been implicated. In men, certain colognes and aftershaves containing lime are suspected triggers [7]. Our patient had a history of regular and prolonged UV exposure due to his regular gardening and long-distance running activities, which may have triggered this condition. The autoimmune hypothesis has also been implicated in LPP. Abnormal T-lymphocyte function and the presence of immunoreactants, like fibrinogen and C3 and immunoglobulin M in colloid bodies, have been identified at the basement membrane zone [4].

The coexistence of LPP with vitiligo has not been well studied. However, the autoimmune hypothesis has also been identified as a probable cause for vitiligo. Humoral immunity of circulating antimelanocyte autoantibodies targeting melanocyte antigens may play a role in its occurrence. The biochemical hypothesis in vitiligo proposes that due to the ultrastructural abnormalities in keratinocytes, there is an increase in H_2_O_2_ that causes cytotoxicity in melanocytes [3]. The theory of melanocytorrhagy and apoptosis proposes that vitiligo is an altered melanocyte response to friction and other types of stress that can cause melanocyte detachment and loss [8]; it could also provide a probable reason for LPP. This further strengthens the finding of LPP and vitiligo being triggered by the Koebner phenomenon and sun exposure, which are sources of stress [9]. The obstruction of lymphocytes in the dermoepidermal junction through immunogenic mechanisms and nonspecific deactivation of immunologic responses was also proposed to cause the development of LPP and vitiligo in 1 patient [6]. All these theories accounting for the pathogenesis of LPP and vitiligo may be considered plausible for our patient.

Given the proposed theories, various treatment options targeting the proposed pathologies have been studied. Low-dose isotretinoin has been observed as a promising therapeutic option for stabilizing and lightening pigmentation in LPP, particularly if given at early onset [10,11], which was also noted in this case report. Further studies are needed to examine isotretinoin’s exact mechanism for improving LPP. It is theorized that it may be due to its anti-inflammatory and immunomodulating ability [11]. Our patient was also given topical tacrolimus 0.1% ointment twice a day, which provided appreciable results in 8 weeks. This proved similar to a study where tacrolimus ointment twice a day significantly lightened hyperpigmented areas after an average of 12 weeks [4]. Tacrolimus also effectively treats vitiligo by downregulating proinflammatory cytokines and promoting melanocyte induction [12]. Though its direct effect on LPP remains unknown, its ability to inhibit T-cell activation and proliferation could account for the improvement in LPP lesions, given the proposed pathophysiology of abnormal T-lymphocyte function. As UV exposure is a trigger for both conditions, it is important to reiterate the use of broad-spectrum sunscreen to hasten improvement and prevent further progression of lesions.

Although these conditions are usually seen separately, due to the proposed autoimmune etiology in response to various types of stress or trauma, both may occur in 1 patient.

## Abbreviations

C3: component 3
LPP: lichen planus pigmentosus
